# Does Kinesiophobia Mediate the Relationship between Pain Intensity and Disability in Individuals with Chronic Low-Back Pain and Obesity?

**DOI:** 10.3390/brainsci11060684

**Published:** 2021-05-22

**Authors:** Giorgia Varallo, Federica Scarpina, Emanuele Maria Giusti, Roberto Cattivelli, Anna Guerrini Usubini, Paolo Capodaglio, Gianluca Castelnuovo

**Affiliations:** 1 Istituto Auxologico Italiano IRCCS, Psychology Research Laboratory, San Giuseppe Hospital, 28824 Verbania, Italy; g.varallo@auxologico.it (G.V.); r.cattivelli@auxologico.it (R.C.); anna.guerriniusubini@unicatt.it (A.G.U.); gianluca.castelnuovo@auxologico.it (G.C.); 2 Department of Psychology, Catholic University of Milan, 20123 Milan, Italy; 3 Rita Levi Montalcini Department of Neurosciences, University of Turin, 10124 Turin, Italy; f.scarpina@auxologico.it; 4 Istituto Auxologico Italiano IRCCS, Unit of Neurology and Neurorehabilitation, San Giuseppe Hospital, 28824 Verbania, Italy; 5 Istituto Auxologico Italiano IRCCS, Orthopaedic Rehabilitation Unit and Clinical Lab for Gait Analysis and Posture, San Giuseppe Hospital, 28824 Verbania, Italy; p.capodaglio@auxologico.it; 6 Department of Surgical Sciences, Physical and Rehabilitation Medicine, University of Turin, 10121 Turin, Italy

**Keywords:** chronic low-back pain, disability, kinesiophobia, fear of movement, fear-avoidance model, obesity, rehabilitation, clinical psychology

## Abstract

Individuals suffering from chronic low-back pain and obesity face severe physical and functional limitations. According to the fear-avoidance model, kinesiophobia might play a crucial role in the relationship between pain intensity and disability. Thus, the purpose of this study was to verify the role of kinesiophobia as a mediator in the association between pain intensity and disability in individuals with both chronic low-back pain and obesity. A total of 213 individuals with chronic low-back pain and obesity were included in the study. The level of kinesiophobia, pain intensity and disability were all assessed using self-reported questionnaires. We verified through a simple mediation analysis that kinesiophobia partially mediated the association between pain intensity and disability in our sample. According to our findings, we emphasize the crucial role of kinesiophobia as a psychological factor that should be addressed in chronic low-back pain rehabilitative protocols to reduce disability in individuals with obesity.

## 1. Introduction

Chronic low-back pain (CLBP) occurs when pain lasts for more than three months and is associated with emotional distress, decreased physical functioning, and is not better accounted for by another disease [1]. CLBP is one of the main causes of disability worldwide [2] and its prevalence is increasing, despite different available treatments, such as surgery and pharmacotherapy [3].

The prevalence of CLBP in the general population is directly associated with higher body mass index (BMI), and obesity (defined as BMI ≥ 30 kg/m^2^ [4]) is a risk factor for its development [5,6]. Thus, as the obesity rates rise, so do the rates of musculoskeletal diseases and associated physical disabilities [7,8].

Individuals with CLBP and obesity experience significant functional limitations and disability [9,10]. They do, indeed, face a dual problem: movement difficulties caused by excess weight, as well as pain interference in daily life due to CLBP [11]. Both conditions have an impact on one another. Thus, reduced physical activity due to pain can result in weight gain [12,13].

The intensity of pain plays a role in determining the level of disability in acute low-back [14,15] and chronic low-back pain sufferers [16]. However, the level of functional disability experienced by people with acute low-back and chronic low-back pain is not entirely explained by the pain intensity level [14,15,16].

Recognizing which factors intervene in the relationship between pain intensity and disability may have a significant impact on how clinicians plan rehabilitative interventions, especially which rehabilitation targets should be prioritized. Nonetheless, the pathways by which pain leads to disability in people with obesity and CLBP remain still unknown [17,18].

According to the bio-psycho-social model, both pain intensity, and biomedical and psychological factors converge to cause functional disability. The fear-avoidance model (FAM) [19,20,21] has become increasingly important in this clinical field. This model underlines the role of cognitive-behavioral factors in the onset of chronic pain. Indeed, according to the FAM [19,20,21] pain intensity and disability are linked via psychological processes, such as pain catastrophizing and fear of pain associated with movements (i.e., kinesiophobia) [19,20,22].

Kinesiophobia is an exaggerated, irrational fear of movement and activities, caused by the perception of vulnerability as a result of a painful injury or fear of reinjury [23]. This fear can lead chronic pain sufferers to avoid activities and movements that they associate with the occurrence or exacerbation of pain [21,24], resulting in deconditioning and disuse [19,25].

In individuals with chronic pain, kinesiophobia is consistently associated with pain intensity [26] and disability [27]. According to emerging research, kinesiophobia is a factor associated with pain intensity [28,29] and disability [29,30] also in individuals with both obesity and CLBP. Moreover, they report suffering from higher levels of kinesiophobia, as well as higher perceived disability and greater mobility impairment than their normal-weight counterparts [12]. Indeed, individuals with obesity frequently complain about dyspnea, musculoskeletal discomfort, and joint pain during physical activity [31], which may make them perceive the physical activity as even more unpleasant and may alter the individual perception of the benefits of movement and physical activity.

However, it appears that kinesiophobia is not only associated with pain intensity [26,29] and disability [27,29] but is also an intervening variable that explains their relationship. Indeed, the FAM [19,20,21] suggests that pain intensity influences disability via the mechanism of kinesiophobia. Previous cross-sectional mediation studies on chronic low-back pain sufferers [16,32] and individuals with a whiplash injury [33] appear to confirm that kinesiophobia plays the role of mediator between pain intensity and disability.

Thus, our research aims to address the following question: is kinesiophobia a psychological factor that can explain how pain intensity causes disability in individuals with CLBP and obesity?

We conducted this cross-sectional study to address this question with the aim of verifying the mediating role of kinesiophobia. As suggested by previous findings relative to chronic back-pain sufferers [16,32] we hypothesized that in people with obesity and CLBP, kinesiophobia would partially mediate the association between pain intensity and disability.

## 2. Materials and Methods

The Ethical Committee of Istituto Auxologico Italiano (code 2020_02_18_04) approved this study. An informed consent document was read, understood, and signed by all participants. All procedures were carried out in line with the Helsinki Declaration of 1975, as revised in 1983.

### 2.1. Participants

A cross-sectional study was carried out. Participants were consecutively recruited, from 1 December 2019 to 28 February 2020, at the Istituto Auxologico Italiano IRCCS, U.O. di Riabilitazione Osteoarticolare, Ospedale S. Giuseppe, Piancavallo, Italia, at the beginning of a month-long hospitalization for weight loss and physical rehabilitation,

Inclusion criteria were as follows: age in years >18 and ≤65; obesity, as measured by a body mass index (i.e., BMI computed as the weight in kilograms divided by the square of height in meters: kg/m^2^) ≥ 30 [4] and CLBP, defined as low back pain duration > three months [1], diagnosed by a rheumatologist at the beginning of the hospitalization.

Exclusion criteria included physical or mental inability to provide signed informed consent; pain duration < three months; diagnosis of another disease that might explain lumbar pain; diagnosis of fracture, neoplasia, bone metastasis, stenosis, which might explain the low back pain; postoperative pain; neurogenic or radicular condition; neurological disease.

Demographic and clinical data were collected through a self-report form completed at the beginning of hospitalization. The completion of the psychological questionnaire was supervised by a researcher.

### 2.2. Measures

The level of disability was measured using the Italian version [34] of the Roland-Morris Disability Questionnaire (RMDQ) [35]. The RMDQ consists of 24 dichotomous items covering daily activities and asks participants to rate their level of difficulty in performing them. A total score ranging from 0 to 24 is computed. Higher levels of pain-related disability are reflected by higher scores. The Italian version of the RMDQ showed levels of reliability and validity comparable to the original version [34]. In the current study, the internal consistency was good (Cronbach’s α = 0.82).

The Numeric Pain Rating Scale (NPRS) [36] was used to evaluate pain intensity levels. The NPRS is composed of an 11-point scale (anchors 0 = no pain, 10 = worst possible pain). The NPRS is a well-validated and widely used measure in chronic pain conditions [37].

To assess the level of kinesiophobia, we used the Italian version [38] of the Tampa Scale of Kinesiophobia (TSK) [38]. The TSK consists of 13 items that range from ‘’strongly agree’’ to ‘’strongly disagree’’ on a 4-point Likert scale [39]. The TSK has two subscales relative to activity avoidance (i.e., belief that pain-inducing activities should be avoided) and harm (i.e., belief that pain is a sign of bodily harm). The total score ranges from 13 to 52; higher scores indicating higher levels of kinesiophobia [38]. The TSK has been validated in CLBP [23]. The Italian version of the TSK shows a good factorial structure and acceptable psychometric properties [38]. In the current sample, the internal consistency of this measure was excellent (Cronbach’s α = 0.90).

### 2.3. Statistical Analysis

Descriptive statistics were calculated in terms of means, standard deviations, and ranges for continuous variables and frequencies and percentages for categorical variables.

Preliminarily, Pearson’s correlation was used to verify the relationship between age, BMI, and scores at NPRS, TSK, and RMDQ; whereas the point-biserial correlation was used to study the association between age, BMI, scores at NPRS, TSK, RMDQ, and sex. Correlation coefficients were classified according to Cohen [40] (0.10 = small; 0.30 = medium; 0.50 = large). Variables with high correlation indicating multicollinearity issues (r > 0.90), or variables that were not correlated with either pain intensity or disability, were excluded from the following mediation analyses.

A simple mediation analysis was carried out. Mediation analysis is a method of investigating an explanatory mechanism by determining the extent to which an intermediary variable (i.e., mediator) explains the association between a predictor and outcome [41].

**Figure 1:**
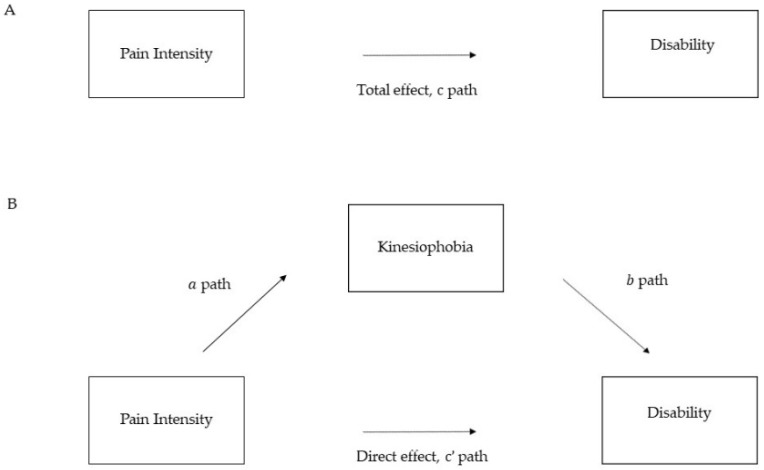
Conceptual diagram of the mediation model. Note. (**A**) The total effect is the effect of pain intensity on disability (c). (**B**) The direct effect (c’) is the effect of pain on disability after controlling for the mediator variables. ‘a path’ is the association between pain intensity and kinesiophobia. ‘b path’ is the association between kinesiophobia and disability controlling for pain intensity.

In our model, kinesiophobia (M) was entered as a mediator in the association between pain intensity (i.e., predictor, X) and the level of disability (i.e., outcome, Y), see Figure 1.

According to Baron and Kenny [42], four conditions had to be met to confirm mediation: (i) pain intensity should be related to disability (total effect; c path = c’ + a × b); (ii) pain intensity should be related to kinesiophobia (a path); (iii) controlling for pain intensity, kinesiophobia should be significantly associated with disability (b path); (iv) the relationship between pain intensity and disability should be reduced (direct effect, c’ path) when controlling for kinesiophobia (indirect effect, a × b).

The simple mediation model was tested using Jamovi 1.2 [43]. We used bias-corrected bootstrap confidence intervals (CI-BC) for inference about indirect effects that it does not require the assumption of normality and reduce the type I error [44,45]. An estimate of the indirect effect was obtained from the mean of 5000 bootstrap samples and 95% CI-BC. The indirect effect is considered statistically significant when confidence intervals (CI-BC) do not include zero.

According to Fritz and Mackinnon [46], our sample size is sufficient for a mediated effect including small-to-medium (0.26) a and b paths with a 0.80 power.

## 3. Results

### 3.1. Participants’ Characteristics

Overall, two hundred and thirteen individuals were included in this study. Table 1 shows the demographic and clinical characteristics of participants; also, we report means, standard deviations, and ranges, for the three main measures (i.e., NPRS: Numeric Pain Rating Scale; TSK: Tampa Scale of Kinesiphobia; RMDQ: Roland Morris Disability Questionnaire).

### 3.2. Preliminary Data Analysis

There were no outliers or missing data. All correlation coefficients between NPRS, TSK, and RMDQ were less than 0.90 indicating the absence of multicollinearity [47]. According to the correlational analyses, age, sex, and BMI, were not significantly correlated with neither NPRS scores nor RMDQ scores (Table 2). Thus, these factors were not included in the mediation model [47].

### 3.3. Mediation Analysis

There was a significant total effect of the level of pain intensity on the level of disability ((i), total effect; c path), (b = 0.669, SE = 0.157, *p* < 0.001). Path a ((ii), association between pain intensity and kinesiophobia), (b = 0.931, SE = 0.223, *p* < 0.001, 95% BC-CI: 0.499, 1.360) and path b ((iii); association between kinesiophobia and disability), (b = 0.236, SE = 0.049, *p* < 0.001, 95% BC-CI: 0.137, 0.330) were significant. The indirect effect via kinesiophobia (iv; a × b path) was significant (b = 0.220, SE = 0.075, *p* = 0.002, 95% BC-CI: 0.094, 0.377). The direct effect ((iv); c’) was reduced compared to the total effect (c), but remained significant (b = 0.449, SE = 0.161, *p* = 0.005).

Overall, these findings revealed that the relationships between the level of pain intensity and the level of pain-related disability were partially mediated by the level of kinesiophobia. Results are reported in Table 3.

## 4. Discussion

As previously reported [28,29,30], kinesiophobia is linked with both pain intensity [28,29] and disability [29,30] CLPB sufferers with obesity. This study expanded on previous findings by verifying the role of kinesiophobia as a mediator. Specifically, we hypothesized that kinesiophobia partially mediates the association between pain intensity and disability.

Our findings appeared to support our hypothesis relative to a partial mediation of kinesiophobia in the association between pain intensity and disability. The results were consistent with previous cross-sectional studies [16,32,48] that investigated this relationship in individuals with whiplash-associated disorders and CLBP, and they bring further evidence in favor of the FAM [19,21].

Our findings broaden our understanding of the psychological factors that contribute to disability. As recently discussed, [49,50], focusing predominately on pain intensity might not be beneficial in the treatment of chronic pain. Indeed, pain might be considered a necessary but not sufficient condition for chronic disability, because not all individuals experiencing CLBP become chronically disabled. Our finding that kinesiophobia plays a role as a mediator in this relationship may help to clarify the circumstances under which disability develops. Experiencing pain can cause fear of injury and movement. resulting in avoidance and inactivity, perceived as protective behaviors, which in turn increases functional restrictions in daily life [20]. Aside from the role of pain intensity per se, the mediating effect of kinesiophobia suggested that how a person reacts to the pain experience (in this case with fear of movement and re-injury) may play a role in disability. Furthermore, kinesiophobia prevents the individual from confronting pain and exposing themselves to fear-inducing movement. Indeed, the FAM [20] hypothesizes confrontation, as opposed to avoidance, as a functional pain-coping strategy that leads to fear reduction in the individual. Taken together, our findings and previous research [14,51] indicate that kinesiophobia is a potential contributor to disability and might be one of the underlying mechanisms explaining how pain leads to disability.

Several clinical implications must be addressed. Developing more tailored treatments for CLBP requires a clarification of the mechanisms that lead to the onset, maintenance of disability. We suggested addressing kinesiophobia in therapeutic interventions especially in the case of associated obesity since individuals suffering from obesity and CLBP might not fully engage in treatments (e.g., physical therapy and exercise). During supervised rehabilitation, gradual exposure to movements that the patient associates with fear might be beneficial to reduce kinesiophobia [52]. A multidisciplinary team comprised of physicians, physical therapists, and psychologists might assist patients through the physical and psychological transition from living in a “pain-restricted” to a “pain-managed” state [30].

Some limitations should be addressed. Due to the cross-sectional design, causality could not be tested. Longitudinal studies should be done to overcome such a limitation. Additionally, since we recruited a care-seeking population recruited from a single center, our findings might not be generalizable to the overall population of individuals with obesity and CLBP. Because our findings support a partial mediation, the presence of other mediators that were not considered in this study should be considered and further examined. Moreover, unexpectedly no significant correlation was observed between pain intensity, disability, and BMI. Even though this result is not in line with some previous evidence [5,6], other studies found no relationship between BMI, pain intensity, and disability [53,54]. Research has suggested that BMI may be an oversimplified measurement of obesity [9,55]. For example, adiposity and the distribution of adipose tissue may be more useful in capturing the condition of obesity [9,56,57].

Despite these limitations, this was the first study investigating the role of kinesiophobia in people with obesity and CBPL. Furthermore, this study was planned to comply with the quality recommendations for mediation analyses [14].

Future research could assess how much kinesiphobia affects behavior by using objective measures, such as the actual level of physical activity levels measured through a pedometer or clinical tests such as the six-minute walking test [58]. It might be also interesting to investigate the mediating effect of kinesiophobia at different levels of BMI and evaluate its potential role as a moderator.

## 5. Conclusions

The current study improves the current knowledge on the psychological factors that contribute to disability. The findings revealed that in individuals with CLBP and obesity, kinesiophobia mediated the association between pain intensity and disability. Our results highlighted the importance of kinesiophobia as factors that should be evaluated and targeted in rehabilitation interventions to reduce disability in CLBP associated whit obesity.

## Figures and Tables

**Table 1:** Demographic and clinical characteristics of the sample (*n* = 213).

	*n* (%)	Mean ± sd	Range (Min-Max)
Sex	M = 68 (31.9); F = 145 (68.1)		
Age (in years)		56.8 ± (9.93)	26–65
BMI (Kg/m^2^)		44.9 ± (8.81)	30–60
NPRS		6.21 ± (2.33)	2–10
TSK		30.4 ± (7.72)	13–50
RMDQ		12.5 ± (5.68)	3–24

Note. M = Male; F = Female; BMI: Body Mass Index; NPRS: Numeric Pain Rating Scale; TSK: Tampa Scale of Kinesiphobia; RMDQ: Roland Morris Disability Questionnaire.

**Table 2:** Pearson and point-biserial correlations coefficients between age, sex and BMI with the measures of pain intensity, kinesiophobia and disability.

	Age (in Years)	Sex	BMI	NPRS	TSK	RMDQ
Age (in years)	-					
Sex	0.058	-				
BMI (Kg/m^2^)	0.110	−0.035	-			
NPRS	0.057	−0.110	−0.035	-		
TSK	0.055	0.076	0.006	0.281 ***	-	
RMDQ	0.048	−0.126	0.131	0.275 ***	0.373 ***	-

Note. BMI: Body Mass Index; NPRS: Numeric Pain Rating Scale; TSK: Tampa Scale of Kinesiphobia; RMDQ: Roland Morris Disability Questionnaire. * *p* < 0.05, ** *p* < 0.01, *** *p* < 0.001.

**Table 3:** Results of the simple mediation analysis.

**Path Estimates**						
	**b**	**SE**	**LLCI**	**ULCI**	**Z**	***p*-Value**
Effect of pain intensity on kinesiophobia (a path)	0.931	0.223	0.499	1.360	4.17	<0.001 ***
Effects of kinesiophobia on disability (b path)	0.236	0.049	0.137	0.330	4.77	<0.001 ***
Effect of pain intensity on disability (c’)	0.449	0.160	0.126	0.759	2.79	0.005 **
**Mediation Estimates**						
	**b**	**SE**	**LLCI**	**ULCI**	**Z**	***p*-Value**
Indirect effect of pain intensity on disability through kinesiophobia (a × b path)	0.220	0.073	0.094	0.377	3.03	0.002 **
Total effect of pain intensity on disability through kinesiophobia (c’ + a × b)	0.669	0.157	0.356	0.966	4.25	<0.001 ***

* *p* < 0.05, ** *p* < 0.01, *** *p* < 0.001.

## Data Availability

The data presented in this study are available on request from the corresponding author. The data are not publicly available due to their containing information that could compromise the privacy of research participants.
